# Supplementary material for: A pan-cancer atlas of therapeutic T cell targets
Source: bioRxiv. 2025 Jul 7:2025.01.22.634237. Preprint. [Version 2] doi: 10.1101/2025.01.22.634237 (PMC12265682; doi:10.1101/2025.01.22.634237)
Supplement: Supplement 10 [file NIHPP2025.01.22.634237v2-supplement-10.pdf]

bioRxiv preprint doi: https://doi.org/10.1101/2025.01.22.634237; this version posted July 7, 2025. The copyright holder for this preprint (which was not certified by peer review) is the author/funder, who has granted bioRxiv a license to display the preprint in perpetuity. It is made available under aCC-BY 4.0 International license.

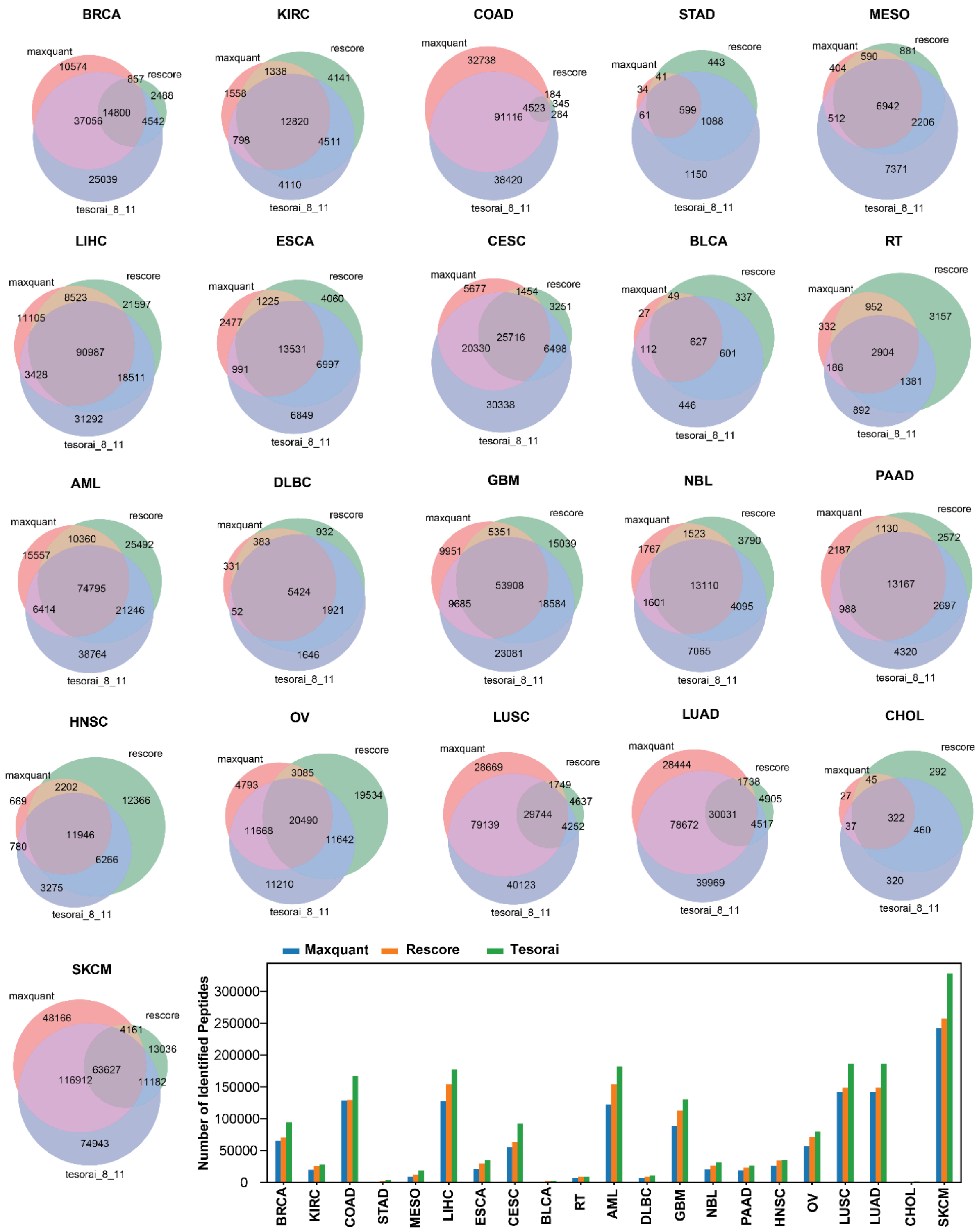


**Supplementary Figure 1. Comparison of identified peptides across MaxQuant, MS2Rescore, and Tesorai Search results across 21 cancers**. Venn diagrams illustrate the

bioRxiv preprint doi: https://doi.org/10.1101/2025.01.22.634237; this version posted July 7, 2025. The copyright holder for this preprint (which was not certified by peer review) is the author/funder, who has granted bioRxiv a license to display the preprint in perpetuity. It is made available under aCC-BY 4.0 International license.

overlap and unique peptides identified by each algorithm across different cancer types. Barplot summarizes the total number of identified peptides per cancer type, highlighting the relative increase achieved by MS2Rescore and Tesorai Search compared to MaxQuant as baseline.

bioRxiv preprint doi: https://doi.org/10.1101/2025.01.22.634237; this version posted July 7, 2025. The copyright holder for this preprint (which was not certified by peer review) is the author/funder, who has granted bioRxiv a license to display the preprint in perpetuity. It is made available under aCC-BY 4.0 International license.

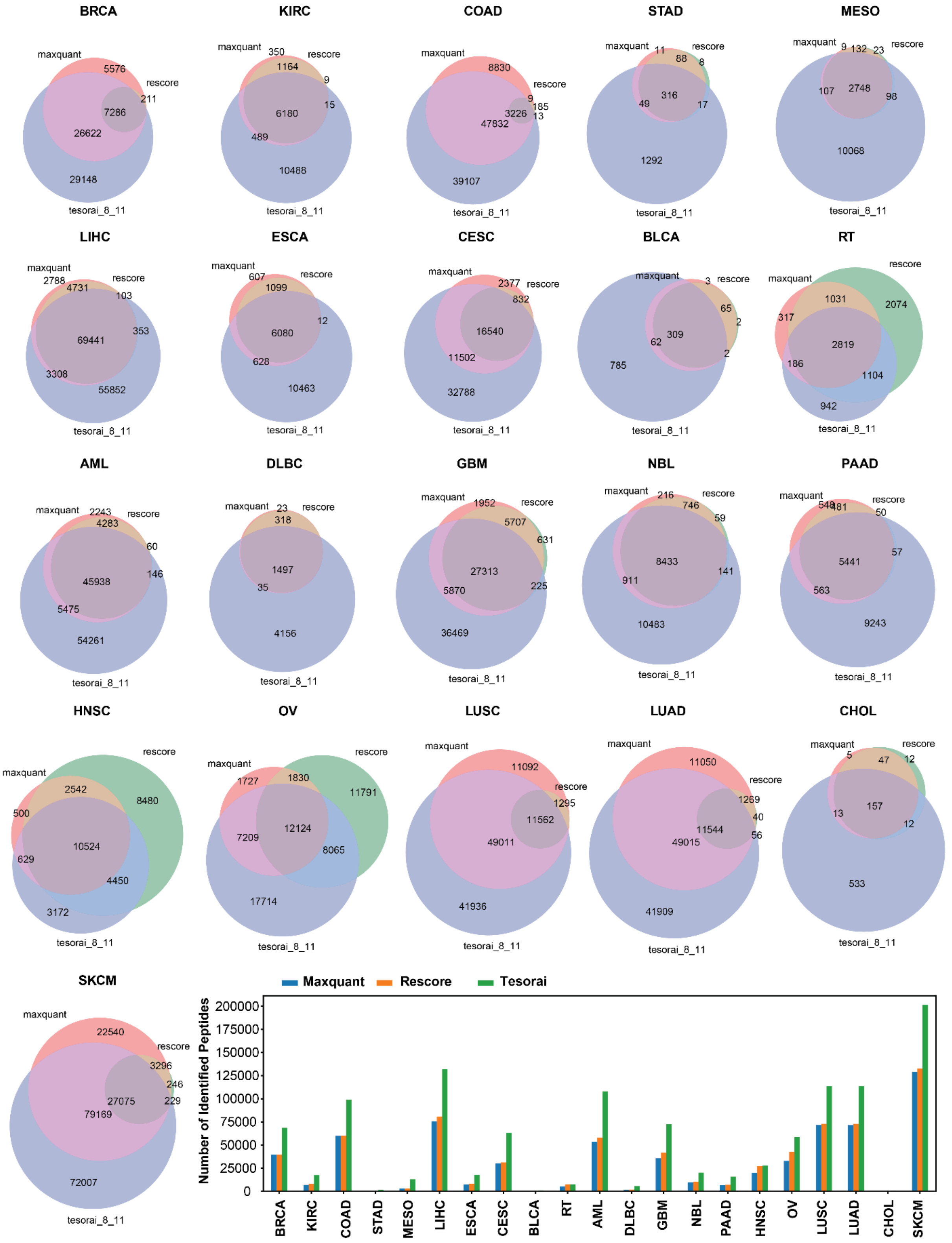


**Supplementary Figure 2. Comparison of identified peptides with high-confidence across**

bioRxiv preprint doi: https://doi.org/10.1101/2025.01.22.634237; this version posted July 7, 2025. The copyright holder for this preprint (which was not certified by peer review) is the author/funder, who has granted bioRxiv a license to display the preprint in perpetuity. It is made available under aCC-BY 4.0 International license.

**MaxQuant, MS2Rescore, and Tesorai search results**. Venn diagrams illustrate the overlap and unique peptides identified by each algorithm across different cancer types. Andromeda score > 70 and Tesorai Score > 5 were used to determine high-confidence peptides after consulting with the original developers. Barplot summarizes the total number of identified peptides per cancer type, highlighting the relative increase achieved by MS2Rescore and Tesorai Search compared to MaxQuant as baseline.

bioRxiv preprint doi: https://doi.org/10.1101/2025.01.22.634237; this version posted July 7, 2025. The copyright holder for this preprint (which was not certified by peer review) is the author/funder, who has granted bioRxiv a license to display the preprint in perpetuity. It is made available under aCC-BY 4.0 International license.

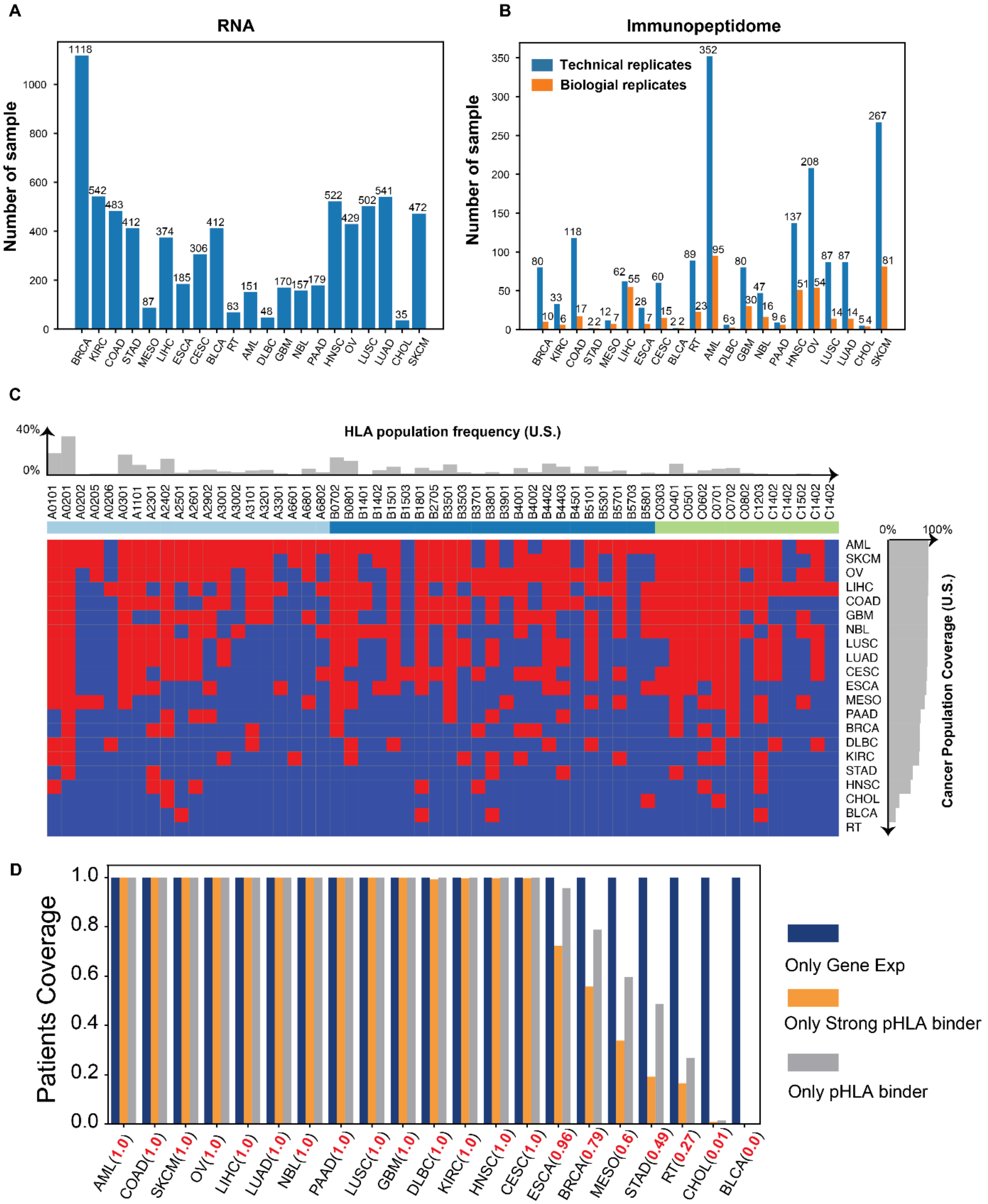


**Supplementary Figure 3. Key statistics associated with public RNA-Seq and immunopeptidome datasets.** (A) Number of RNA-Seq datasets for each tumor across TCGA and TARGET. (B) Number of immunopeptidomic datasets for each tumor collected throughout

bioRxiv preprint doi: https://doi.org/10.1101/2025.01.22.634237; this version posted July 7, 2025. The copyright holder for this preprint (which was not certified by peer review) is the author/funder, who has granted bioRxiv a license to display the preprint in perpetuity. It is made available under aCC-BY 4.0 International license.

public repositories, number of technical and biological replicates were annotated (C) HLA coverage across cancer types. Bar plots display common HLA alleles (with U.S. population frequency >1%) and their corresponding population coverage, grouped by HLA-A, HLA-B, and HLA-C categories. Red and blue indicate the presence and absence, respectively, of the HLA allele in each cancer cohort. (D) Patient populations potentially eligible for PC-CAR therapy, as evaluated based on gene expression alone and further stratified by tumor-specific HLA restrictions. Both strong and weak peptide-HLA binders were considered in the analysis.

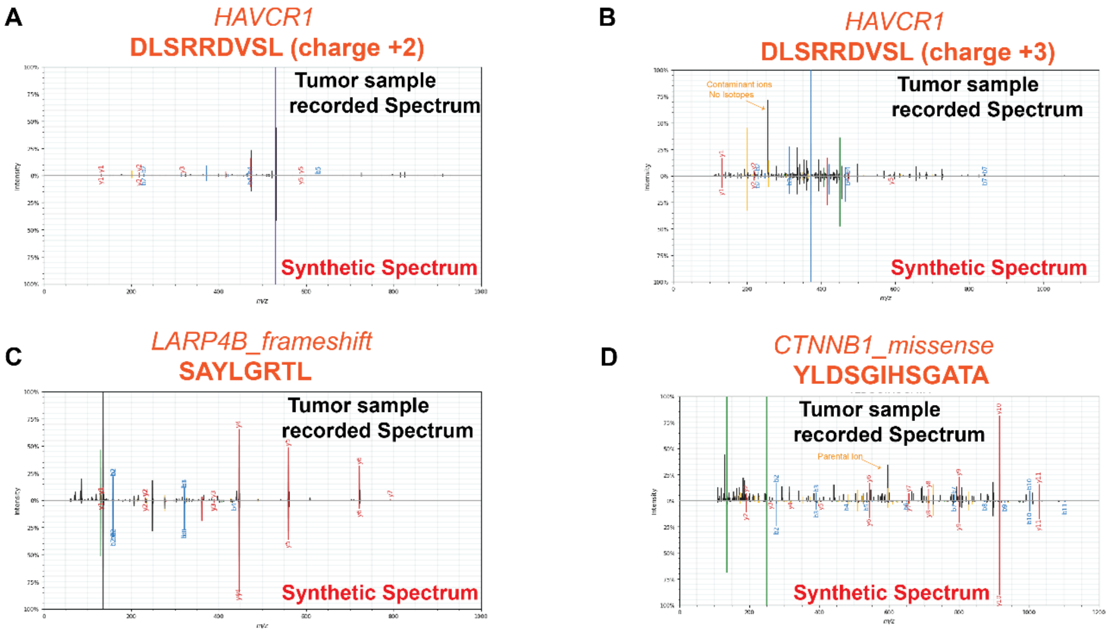


**Supplementary Figure 4. Spike-in mass spectrometry validation of HAVCR1 peptides and mutation-derived neoantigens.** (A–B) Mirror plots for HAVCR1 peptides at charge states +2 and +3, respectively, representing the highest-scoring spectra identified by MaxQuant and Tesorai Search, compared to corresponding synthetic spectrum. (C) Mirror plot for the frameshift

bioRxiv preprint doi: https://doi.org/10.1101/2025.01.22.634237; this version posted July 7, 2025. The copyright holder for this preprint (which was not certified by peer review) is the author/funder, who has granted bioRxiv a license to display the preprint in perpetuity. It is made available under aCC-BY 4.0 International license.

mutation–derived neoantigen SAYLGRTL from LARP4B. (D) Mirror plot for the missense mutation–derived neoantigen YLDSGIHSGATA from CTNNB1.

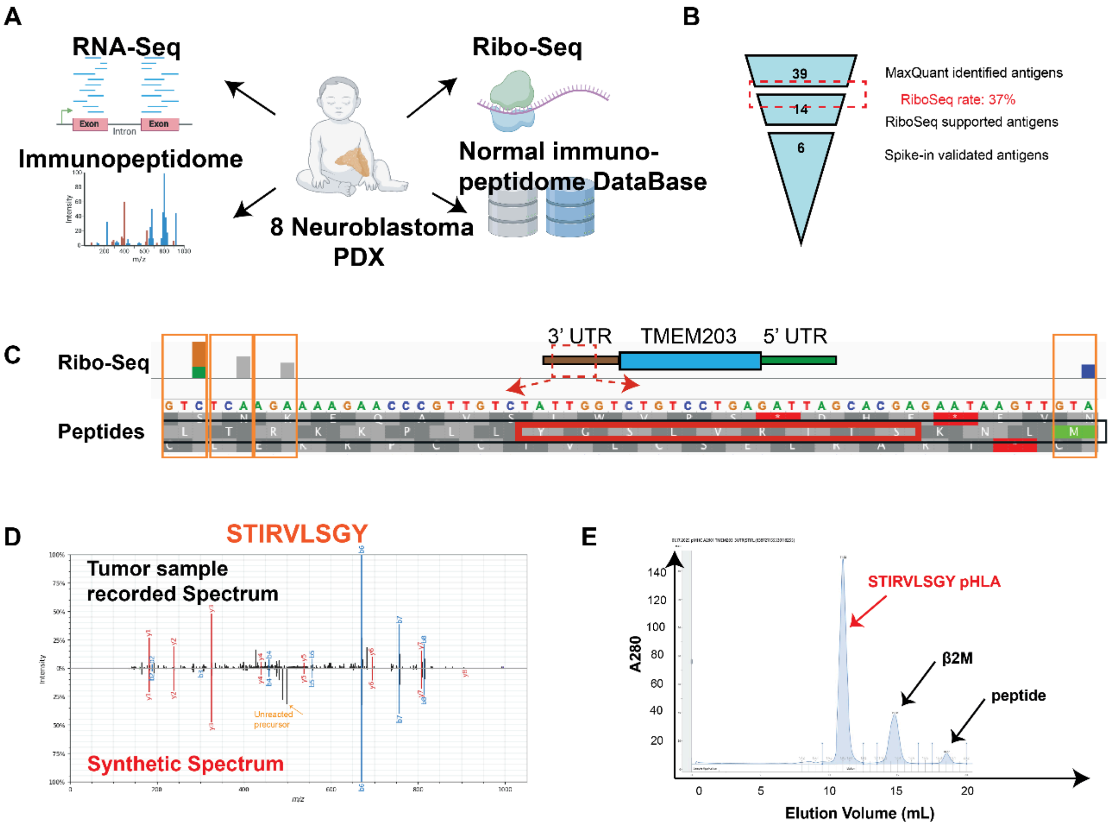


**Supplementary Figure 5. Cryptic ORF antigen validation workflow and TMEM203 3' UTR derived STIRVLSGY antigen.** (A) workflow of assessing translational activity of cryptic ORFs using Ribo-Seq in matched PDX neuroblastoma models (B) funnel plot of validated cryptic ORF antigen in matched Neuroblastoma PDX model based on Ribo-Seq evidence (C) Ribosome occupancies on 3' UTR of TMEM203 from Ribo-Seq, the STIRVLSGY peptide (residing on negative strand) was highlighted in red box and the Ribo-Seq confirmed codons were

bioRxiv preprint doi: https://doi.org/10.1101/2025.01.22.634237; this version posted July 7, 2025. The copyright holder for this preprint (which was not certified by peer review) is the author/funder, who has granted bioRxiv a license to display the preprint in perpetuity. It is made available under aCC-BY 4.0 International license.

highlighted in orange box  (D) Mirror plot of synthetic peptide and original spectra to confirm the Mass Spectrometry (MS) identification of TMEM203 cryptic ORF antigen (E) Size exclusion chromatography after refolding the STIRVLSGY-HLA-A*26:01 complex.

bioRxiv preprint doi: https://doi.org/10.1101/2025.01.22.634237; this version posted July 7, 2025. The copyright holder for this preprint (which was not certified by peer review) is the author/funder, who has granted bioRxiv a license to display the preprint in perpetuity. It is made available under aCC-BY 4.0 International license.

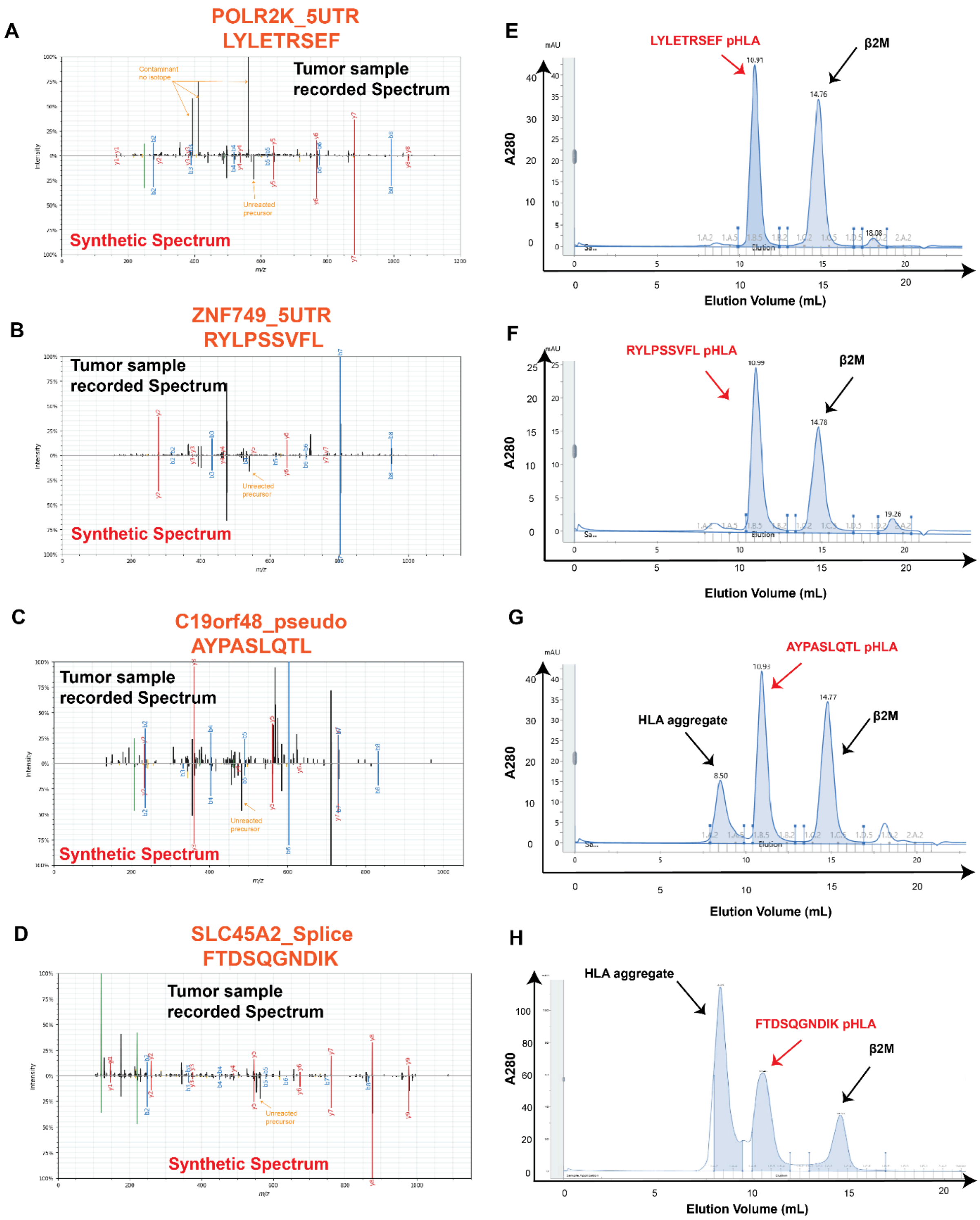


**Supplementary Figure 6. Validation of additional Cryptic ORF and splicing antigens.** (A-D)

Mirror plots of original spectrum from the tumor samples and the synthetic peptide spectra to

bioRxiv preprint doi: https://doi.org/10.1101/2025.01.22.634237; this version posted July 7, 2025. The copyright holder for this preprint (which was not certified by peer review) is the author/funder, who has granted bioRxiv a license to display the preprint in perpetuity. It is made available under aCC-BY 4.0 International license.

validate the MS identification for POLR2K_5UTR, ZNF749_5UTR, C19orf48_pseudogene, SLC45A2_spliced (top to bottom). (E-H) Size Exclusion Chromatography after refolding pHLA for LYLETRSEF-A2402 complex, RYLPSSVFL-A2402 complex, AYPASLQTL-A2402, FTDSQGNDIK-A1101 complex (top to bottom).

bioRxiv preprint doi: https://doi.org/10.1101/2025.01.22.634237; this version posted July 7, 2025. The copyright holder for this preprint (which was not certified by peer review) is the author/funder, who has granted bioRxiv a license to display the preprint in perpetuity. It is made available under aCC-BY 4.0 International license.

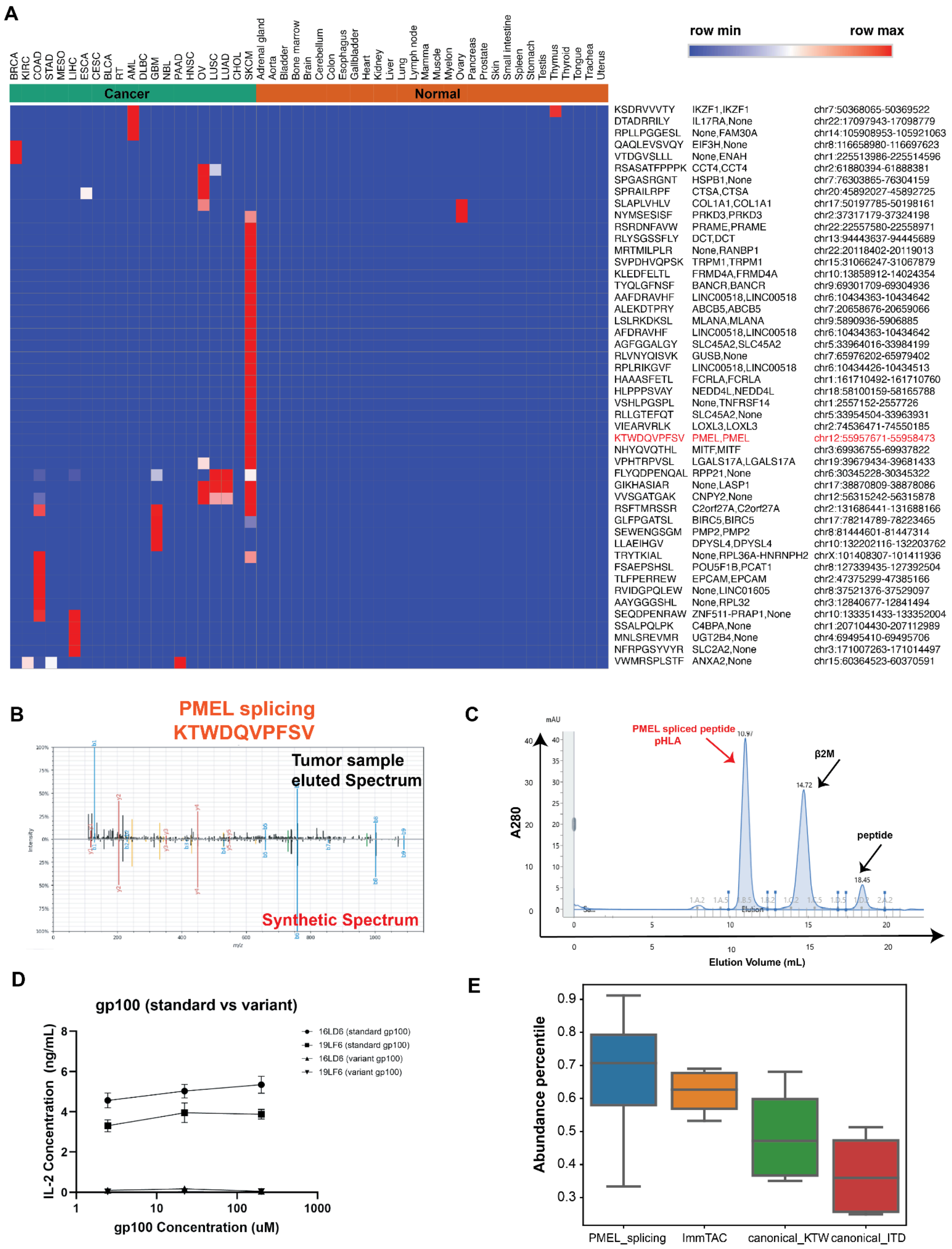


**Supplementary Figure 7. Splicing antigen landscapes across tumor types.** (A)

bioRxiv preprint doi: https://doi.org/10.1101/2025.01.22.634237; this version posted July 7, 2025. The copyright holder for this preprint (which was not certified by peer review) is the author/funder, who has granted bioRxiv a license to display the preprint in perpetuity. It is made available under aCC-BY 4.0 International license.

High-confidence and frequently detected splicing antigen across 21 cancers, annotated by the parental genes of the two splicing sites (splicing acceptor side and splicing donor site, None denotes the splicing sites are unannotated novel splicing sites) and chromosomal coordinates (B) Validation of PMEL spliced peptide using synthetic peptide MS/MS (C) Size exclusion chromatography after refolding PMEL spliced peptide and HLA-A*02:01 allele (D) TCR-T co-culture ELISA assay using specific TCR for canonical ITD antigens toward both canonical ITD and spliced variant (E) Comparison of peptide abundance (expressed as percentile of abundance) amongst four PMEL antigens.

bioRxiv preprint doi: https://doi.org/10.1101/2025.01.22.634237; this version posted July 7, 2025. The copyright holder for this preprint (which was not certified by peer review) is the author/funder, who has granted bioRxiv a license to display the preprint in perpetuity. It is made available under aCC-BY 4.0 International license.

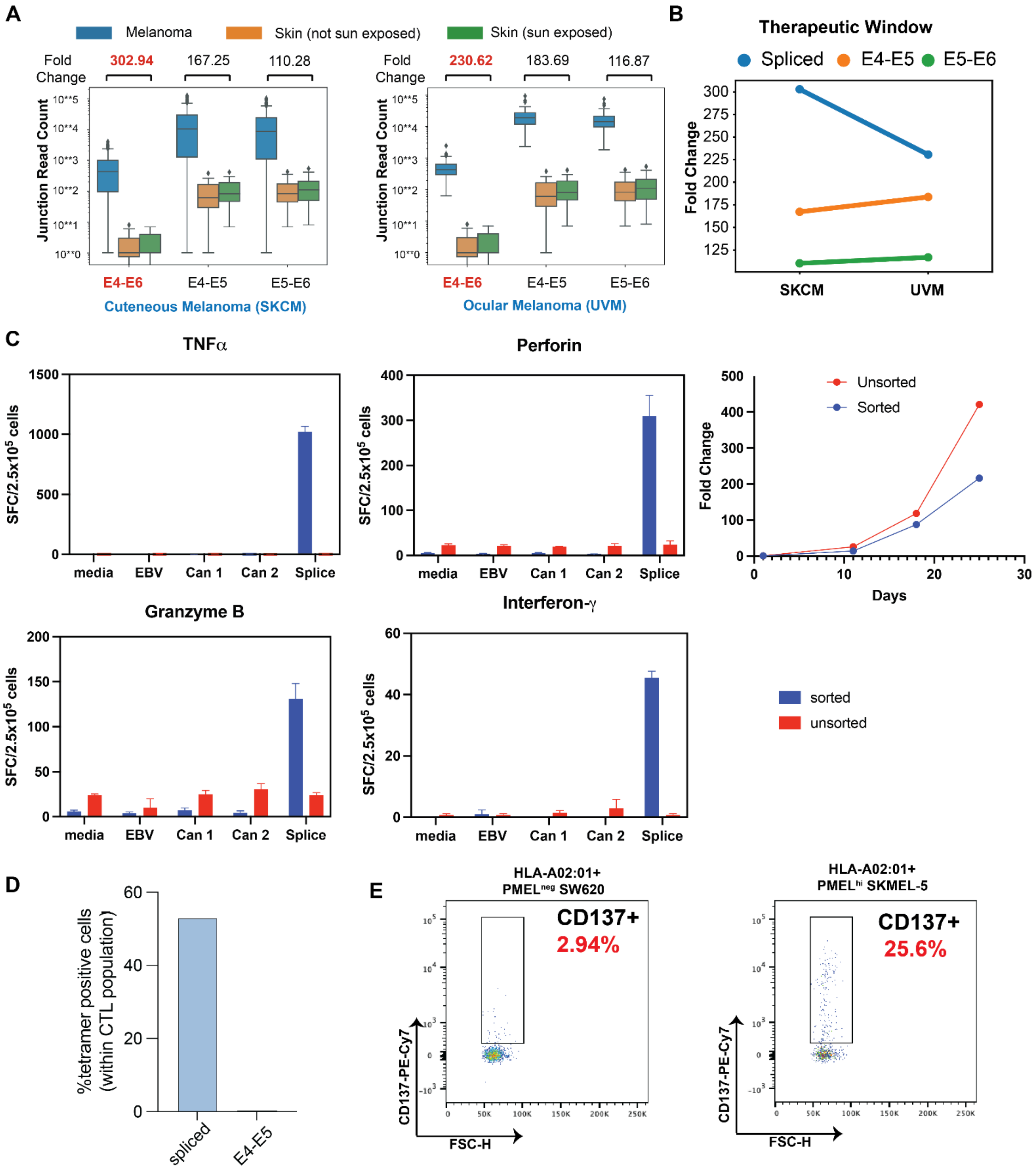


**Supplementary Figure 8. Therapeutic windows of spliced PMEL (sPMEL) and enrichment of sPMEL-specific TCRs.** (A) Therapeutic windows on RNA level between spliced and canonical junctions in both cutaneous melanoma and ocular melanoma. The fold change is derived between melanoma and sun exposed skin (B) The changes of therapeutic windows

bioRxiv preprint doi: https://doi.org/10.1101/2025.01.22.634237; this version posted July 7, 2025. The copyright holder for this preprint (which was not certified by peer review) is the author/funder, who has granted bioRxiv a license to display the preprint in perpetuity. It is made available under aCC-BY 4.0 International license.

between cutaneous melanoma and ocular melanoma for PMEL spliced antigens and two canonical counterparts. (C) sPMEL-tetramer presorted T cells from a healthy donor exhibit specific expansion in response to sPMEL antigen, as compared to control tetramers loaded with the Can1 peptide (E4–E5 junction), Can2 peptide (E5–E6 junction), and a positive control peptide from EBV (CLGGLLTMV). Antigen-specific responses were assessed by measuring secretion levels of TNFα, perforin, granzyme B, and interferon-γ, as well as fold changes in cell numbers over the course of expansion. (D) Binding to spliced-derived peptide as well as canonical (KTWGQYWQV) peptide is quantified within the CTL population. (E) Representative flow plots showing CD137 expression within CTL population without stimulation or 20h after co-culture with HLA-A02:01+Pmel$^{high}$ SKMEL-5 melanoma cell line.

bioRxiv preprint doi: https://doi.org/10.1101/2025.01.22.634237; this version posted July 7, 2025. The copyright holder for this preprint (which was not certified by peer review) is the author/funder, who has granted bioRxiv a license to display the preprint in perpetuity. It is made available under aCC-BY 4.0 International license.

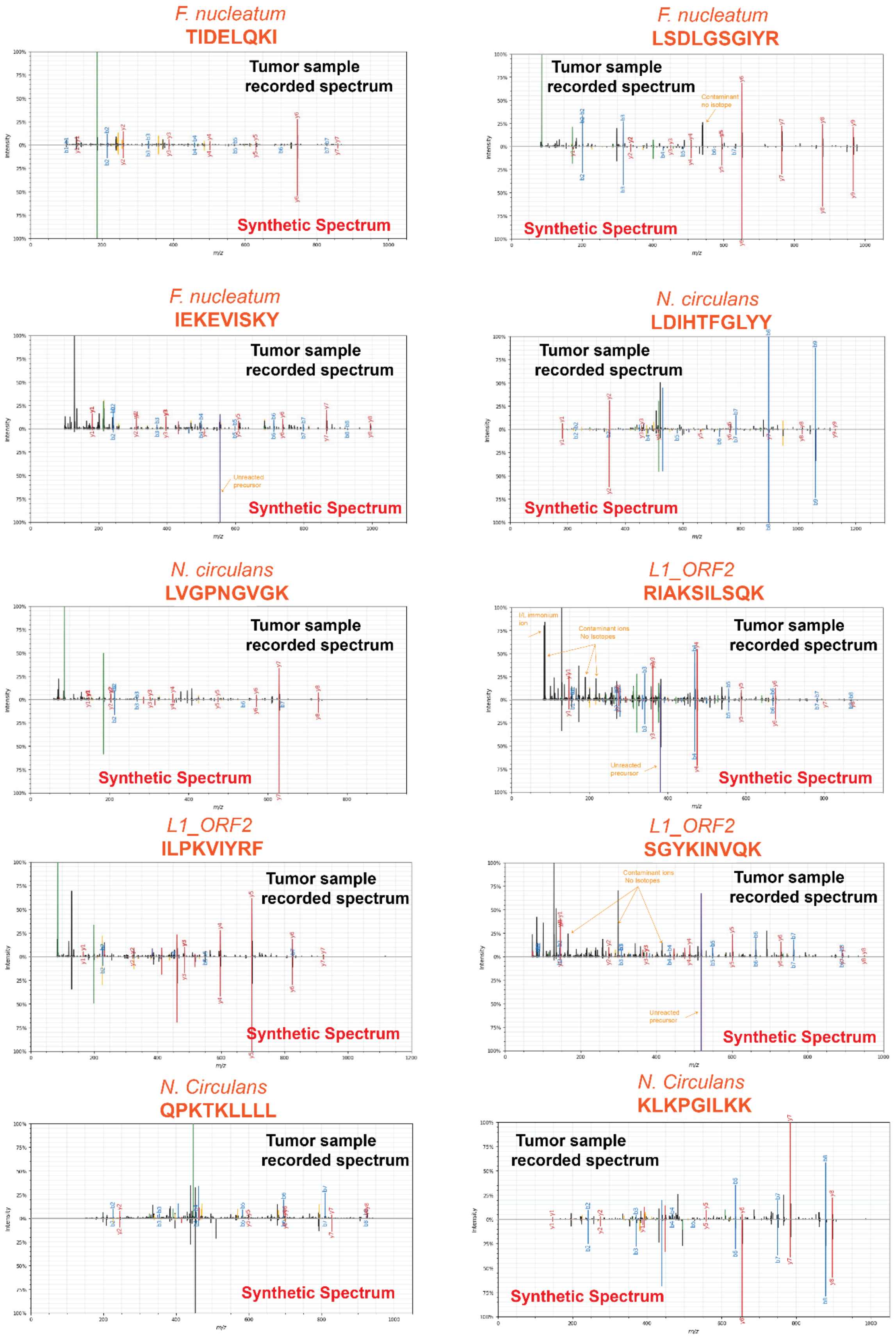

bioRxiv preprint doi: https://doi.org/10.1101/2025.01.22.634237; this version posted July 7, 2025. The copyright holder for this preprint (which was not certified by peer review) is the author/funder, who has granted bioRxiv a license to display the preprint in perpetuity. It is made available under aCC-BY 4.0 International license.

**Supplementary Figure 9. Validation of pathogen and TE derived antigens.** Mirror plots comparing the original MS/MS spectra from tumor samples with the corresponding synthetic peptide spectra to validate the identification of nine pathogen-derived peptides. These include peptides from *Fusobacterium nucleatum* (TIDELQKI, LSDLGSGIYR, IEKEVISKY), *Niallia circulans* (LDIHTFGLYY, LVGPNGVGK), and L1_ORF2 (RIAKSILSQK, ILPKVIYRF, SGYKINVQK). Two additional peptides derived from *Niallia circulans* (QPKTKLLLL and KLKPGILKK), which exhibit high spectral similarity, were acquired in the tumor sample using Wideband Activation mode that is only available for the linear trap quadrupole (LTQ) Orbitrap XL mass spectrometer. This instrument is not available for the analysis of the synthetic peptide, instead, Multistage Activation was used for these two peptides, targeting also the M-17 ion for fragmentation.

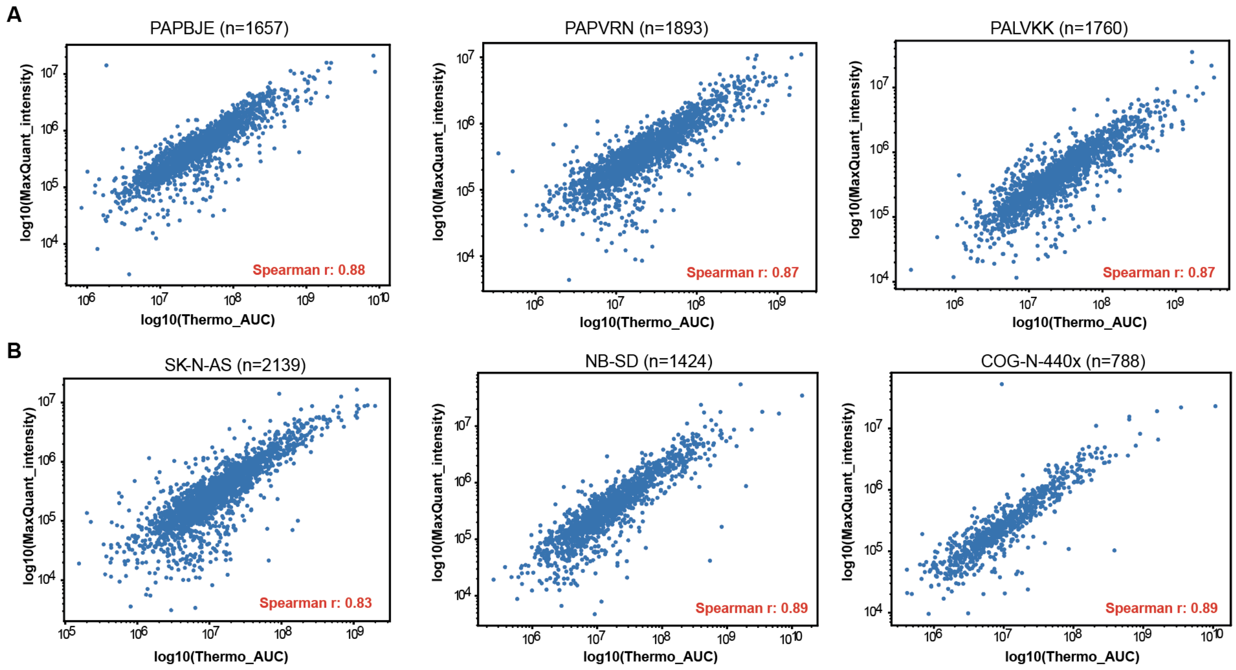


**Supplemental Figure 10. Correlation between maximum precursor intensity reported by MaxQuant and Area Under the Curve (AUC) reported by Thermo proprietary software**

bioRxiv preprint doi: https://doi.org/10.1101/2025.01.22.634237; this version posted July 7, 2025. The copyright holder for this preprint (which was not certified by peer review) is the author/funder, who has granted bioRxiv a license to display the preprint in perpetuity. It is made available under aCC-BY 4.0 International license.

**(ProteomeDiscoverer).** (A) Spearman correlation of intensity values for commonly detected peptides in three neuroblastoma primary tumors (PAPBJE, PAPVRN, PALVKK) with the highest number of overlapping identifications. (B) Spearman correlation of intensity values for commonly detected peptides in three neuroblastoma cell lines (SK-N-AS, NB-SB, COG-N-440x) with the highest overlap.
